# Cutaneous metallosis following ceramic insert fracture in total hip arthroplasty: a case report and revision with ceramic-on-ceramic bearing couple

**DOI:** 10.1051/sicotj/2025007

**Published:** 2025-03-07

**Authors:** Vasileios Giovanoulis, Angelo V. Vasiliadis, Simon Marmor

**Affiliations:** Department of Orthopedic Surgery, Groupe Hospitalier Diaconesses Croix Saint-Simon 125 rue d’Avron 75020 Paris France

**Keywords:** Hip, Metallosis, Ceramic, Bearing, Revision

## Abstract

Ceramic fractures in total hip arthroplasty (THA) are rare complications that pose significant challenges for revision surgery. This case report describes a 68-year-old male who experienced a spontaneous alumina (ceramic) insert and head fracture four years after the initial THA. The first revision with cobalt-chrome and polyethylene components led to severe metallosis, including subcutaneous tissue discoloration. A second revision utilized a ceramic-on-ceramic (CoC) bearing couple, resulting in excellent functional outcomes and resolution of symptoms. Cutaneous pigmentation post-THA is rare and has not been previously reported following a ceramic fracture. The case underscores the need for careful material selection in revision surgery to minimize complications such as metallosis. The decision to use a ceramic-on-ceramic bearing couple in this case proved effective, ensuring durability and reducing the risk of third-body wear, which can result from inadequate management of ceramic fractures and lead to joint, systemic, or cutaneous complications.

## Introduction

Total hip arthroplasty (THA) is among the most successful orthopedic procedures. Surgeons must carefully select bearing surfaces to optimize outcomes [1]. Tribology, the study of friction, lubrication, and wear between moving surfaces, derives from the Greek word “Τριβή”, meaning “rubbing” [1]. Ceramic-on-ceramic (CoC) bearings are associated with minimal wear, excellent biocompatibility, and low complications [1] but pose risks of fracture and noise generation after arthroplasty [1]. Ceramic fractures (CF) are rare but recognized complications in THA. Prompt diagnosis and management are essential, as delayed intervention can lead to catastrophic outcomes [2]. CF can result in significant bone and soft tissue defects or implant destruction due to the abrasive nature of alumina particles, leading to third-body wear [2]. Literature underscores that the highest reported blood levels of chromium and cobalt ions are not absolutely linked to metal-on-metal bearings but instead occur following ceramic fractures, particularly when chromium-cobalt components are used in revision procedures [3].

Skin pigmentation after total hip arthroplasty was exceptionally described after metal-on-metal bearing [2] but to our knowledge, this has never been reported following a ceramic fracture. This case highlights the unique presentation of cutaneous metallosis following a ceramic fracture in total hip arthroplasty [2]. We present the case of a 68-year-old male who experienced a ceramic fracture (CF) four years after a total hip arthroplasty (THA). The initial revision using chrome-cobalt components led to severe metallosis of the hip. A second revision, utilizing a ceramic-on-ceramic (CoC) bearing, successfully resolved the symptoms and restored excellent functional outcomes.

### Statement of informed consent

The patient was informed that data concerning the case would be submitted for publication and agreed.

### Presentation of the case

- February 2009 (Primary THA): A 68-year-old male, with a Body Mass Index of 27 kg/m^2^ and no significant medical history, underwent a primary elective THA for osteoarthritis in the left hip using the posterior (Moore) approach. The implants used were a 58-mm ATLAS™ ^®^ cup featuring an alumina/polyethylene sandwich ceramic insert and an FH Ortho Hip’n Go stem^®^ with a short neck alumina head (Figure 1).
  **Figure 1:**
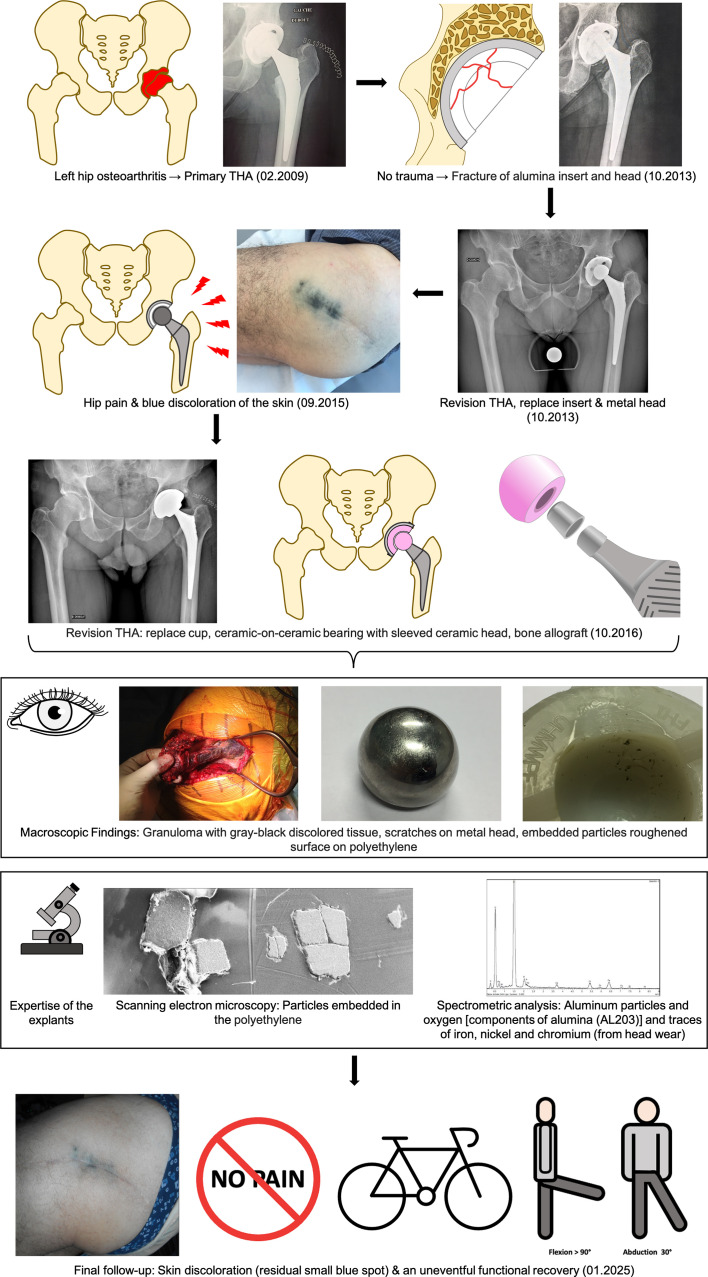
Flowchart of treatment management (abbreviations: THA, total hip arthroplasty; PE, polyethylene).
- 2013 (First revision – Metal-on-Polyethylene): At four years postoperatively, the patient developed a spontaneous, atraumatic fracture of the ceramic insert and femoral head. Radiographs revealed no acetabular bone defects or fractures (Figure 1) and the femoral stem was not loosened. Revision surgery was performed, replacing the insert and head with a metal-polyethylene bearing couple (Figure 1). No postoperative complications were noted, and the patient regained his pre-surgery autonomy. Postoperative radiographs did not show any residual ceramic particles.
- 2015 (Onset of symptoms): In 2015, he presented with hip pain, discomfort, and a marked black discoloration over the lateral scar area (Figure 1). Infection was ruled out through normal blood tests and sterile preoperative joint aspiration.
- October 2016 (Second revision – Ceramic-on-Ceramic): The patient underwent acetabular revision with an uncemented 60-mm Dynacup Corin^®^ cup and alumina insert, along with replacement of the metal head with a Revision Biolox Delta^®^ ceramic head (36 mm, 12/14 taper) (Figure 1). Intraoperative findings included subcutaneous infiltration of gray-black discolored tissues, which were more present near the prosthetic joint (Figure 1). An extensive synovial debridement was performed. Removal of the metal head and polyethylene liner posed no challenges, and the femoral stem was stable. Minimal damage to the femoral neck was observed, and the Morse taper remained intact and undamaged, eliminating the need for stem replacement. Bone loss in the anterior column due to metallosis required reconstruction with morselized lyophilized allograft. Full weight-bearing was allowed immediately postoperatively.

Preoperative and intraoperative bacteriological samples returned sterile results. Macroscopic examination of the retrieved implants showed embedded particles in the polyethylene (PE), a roughened surface on the polyethylene, and significant scratches on the metal head (Figure 1).

Pathological analysis revealed hyperplastic synovium with fibrous changes and a macrophagic reaction to black pigments (indicative of metallosis). Scanning Electron Microscopy (SEM) revealed embedded particles within the polyethylene insert (Figure 1). Spectrometry and X-ray microanalysis identified two primary components of these particles: aluminum and oxygen, which are the main constituents of alumina (Al_2_O_3_). Additionally, traces of iron, nickel, and chromium were detected on these particles, originating from the wear of the stainless steel head (Figure 1).

- At the latest follow-up, eight years post-revision, the patient continues to exhibit excellent clinical outcomes. He remained entirely asymptomatic, with no limitations in daily activities or hip function. Furthermore, he engaged in sports such as cycling and martial arts without any restrictions. The skin discoloration observed postoperatively has remained unchanged, presenting the same minimal pigmentation noted immediately after surgery (Figure 1). The patient presented a Postel Merle d’Aubigné score of 18/18 (6/6/6), reaffirming the long-term success of the surgical approach. A post-operative X-ray at 8 years follow-up is shown in Figure 2 without signs of wear or osteolysis.
  **Figure 2:**
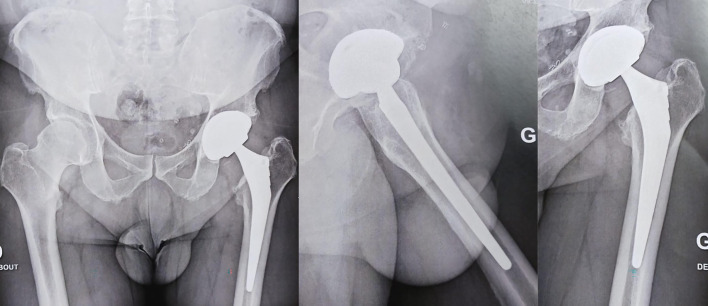
Postoperative X-ray at eight-year follow-up demonstrating implant stability.

## Discussion

This case demonstrates that revising a ceramic fracture with a ceramic-ceramic bearing couple can achieve long-term outcomes, including alleviation of cutaneous symptoms. Cutaneous metallosis and skin pigmentation remain a rare and underdiagnosed complication. It has been reported with metal-on-metal bearings, knee and shoulder arthroplasties, and even simpler procedures like osteosynthesis [1]. Diagnosis may be aided by skin biopsy to detect chromium-cobalt particles. Cutaneous metallosis following the ceramic fracture is scarcely reported in the literature [2]. This case underscores the importance of material selection in revision THA to prevent metallosis.

### Incidence of ceramic fractures

Literature highlights the evolution of ceramic implant design. Hannouche et al. [3] reported eight ceramic fractures (six head fractures and two insert fractures) from 5,500 Biolox Forte^®^ implants between 1977 and 1990. Currently, the alumina matrix composite (AMC) (Biolox Delta; CeramTech AG, Plochingen, Germany) is the ceramic material most widely utilized [1]. These subsequent improvements have dramatically reduced fracture rates to 0.002% in recent years. Liner fractures are rarely caused by direct trauma; instead, they typically result from one of three primary reasons: improper alignment during liner insertion, damage to the metal backing, or malpositioning of the acetabular component, which can cause impingement and edge-loading [1].

### Risk factors for ceramic fractures

Identified risk factors include older-generation ceramics, sandwich designs (e.g., ATLAS cups), malalignment, high BMI, short femoral neck and low ceramic thickness [3].

### Recommendations for revision management

There is no established consensus on the optimal tribology approach for CF revision surgery but it is of paramount importance that the bearing couple selected for revision after a ceramic component failure should not include cobalt-chromium. Metal head-on PE liners can provoke extensive metal wear, leading to severe metallosis and metal toxicity [3, 4]. Hard ceramic particles acting as third-body wear can cause severe damage to the metal head, resulting in some of the highest blood metal ion levels reported in the literature [3]. These elevated levels are not only linked to metal-on-metal bearings but are significantly associated with the use of cobalt-chromium components following ceramic fractures [1]. Such elevated levels have been implicated in systemic complications, including a reported case of fatal cardiomyopathy [5] after the use of a metal-on-PE bearing couple to address a CF. This approach is, therefore, explicitly contraindicated.

### Optimal tribology for CF revision

Zagra and Gallazzi [1] suggest using ceramic on polyethylene bearing couple for revision. There were no instances of re-revision attributed to tribological issues, with only one case requiring further revision due to polyethylene (PE) wear and osteolysis, which was related to improper acetabular cup positioning. Marmor et al. [4] used a ceramic-on-ceramic bearing couple following CF. In a long-term series, no complications or implant failures occurred, and all patients expressed satisfaction and excellent functional scores. No cases of osteolysis or loosening were observed. Furthermore, despite performing extensive synovectomies, residual ceramic particles were detected on radiographs in 50% of cases. This underscores the challenge of achieving complete removal of ceramic debris even with meticulous surgical debridement.

### Clinical takeaway

Surgeons should prioritize ceramic-on-ceramic bearings in revision surgeries for ceramic fractures to ensure durability, minimize complications, and reduce the risk of systemic toxicity and third-body wear. If this is not feasible, a ceramic-on-polyethylene bearing can be used as a degraded alternative [3].

## Conclusion

Our case represents an unusual clinical presentation of skin pigmentation resulting from a ceramic fracture revised with a metal-on-polyethylene bearing couple. Inadequate management of ceramic fractures can lead to joint, systemic, or cutaneous complications. The decision to use a ceramic-ceramic bearing couple, in this case, proved effective, ensuring durability and reducing the risk of third-body wear.

## Data Availability

The Data are available from the corresponding author upon reasonable request.
